# Analysis of transcript changes in a heme-deficient mutant of *Escherichia coli* in response to CORM-3 [Ru(CO)_3_Cl(glycinate)]

**DOI:** 10.1016/j.gdata.2015.06.008

**Published:** 2015-06-13

**Authors:** Jayne Louise Wilson, Samantha McLean, Ronald Begg, Guido Sanguinetti, Robert K. Poole

**Affiliations:** a Department of Molecular Biology and Biotechnology, The University of Sheffield, Sheffield S10 2TN, UK; b School of Informatics, The University of Edinburgh, Edinburgh EH8 9AB, UK

**Keywords:** *Escherichia coli*, Heme deficient mutant, CO-releasing molecule, Transcriptomics, Statistical modelling

## Abstract

This article describes in extended detail the methodology applied for acquisition of transcriptomic data, and subsequent statistical data modelling, published by Wilson *et al*. (2015) in a study of the effects of carbon monoxide-releasing molecule-3 (CORM-3 [Ru(CO)_3_Cl(glycinate)]) on heme-deficient bacteria. The objective was to identify non-heme targets of CORM action. Carbon monoxide (CO) interacts with heme-containing proteins, in particular respiratory cytochromes; however, CORMs have been shown to elicit multifaceted effects in bacteria, suggesting that the compounds may have additional targets. We therefore sought to elucidate the activity of CORM-3, the first water-soluble CORM and one of the most characterised CORMs to date, in bacteria devoid of heme synthesis. Importantly, we also tested inactive CORM-3 (iCORM-3), a ruthenium co-ligand fragment that does not release CO, in order to differentiate between CO- and compound-related effects. A well-established *hemA* mutant of *Escherichia coli* was used for the study and, for comparison, parallel experiments were performed on the corresponding wild-type strain. Global transcriptomic changes induced by CORM-3 and iCORM-3 were evaluated using a Two-Color Microarray-Based Prokaryote Analysis (FairPlay III Labeling) by Agilent Technologies (Inc. 2009). Data acquisition was carried out using Agilent Feature Extraction software (v6.5) and data normalisation, as well as information about gene products and their function was obtained from GeneSpring GX v7.3 (Agilent Technologies). Functional category lists were created using KEGG (Kyoto Encyclopedia of Genes and Genomes). Relevant regulatory proteins for each gene were identified, where available, using regulonDB and EcoCyc (World Wide Web). Statistical data modelling was performed on the gene expression data to infer transcription factor activities. The transcriptomic data can be accessed through NCBI's Gene Expression Omnibus (GEO): series accession number GSE55097 (http://www.ncbi.nlm.nih.gov/geo/query/acc.cgi?acc=GSE55097).

**Table:** 

Specifications	
Organisms	*Escherichia coli* K12 MG1655 (wild-type strain) *Escherichia coli* K12 W3310 *hemA*::Km^R^ (*hemA* mutant strain; Keio collection (1,2)).
Sex	*N*/*A*
Data acquisition	Transcriptomic data were acquired using a ‘Two-Color Microarray-Based Prokaryote Analysis (FairPlay III Labeling)’ by Agilent Technologies (Inc. 2009) and an Agilent DNA microarray scanner (G2505) controlled by Agilent Scan Control software (v8.5).
Data format	Raw
Experimental factors	The *hemA* mutant allele was P1-transduced from *E. coli* strain W3310 into *E. coli* strain MG1655. Global transcriptomic analyses were employed to compare changes in the transcriptome of the *hemA* mutant versus the wild-type following treatment with CORM-3.
Experimental features	Wild-type and *hemA* strains of *E. coli* MG1655 were grown under anaerobiosis in batch culture in custom-made, stirred in 250 ml mini-fermenter vessels at 37 °C during continuous sparging with 100% nitrogen gas. Cells were grown in a defined minimal medium containing 0.5% glucose as the sole and limiting source of energy and carbon. The medium was supplemented with 0.1% casamino acids and 5% LB to support the growth of the *hemA* mutant; kanamycin (50 μg/ml) was added to cultures of the heme-deficient mutant only. At an OD_600_ of 0.2, CORM-3 (100 μM) was added to wild-type and mutant cultures, and iCORM-3 (100 μM) was added to mutant cultures only. Culture samples were taken immediately prior to the addition of compounds and at 10, 20, 40, 60 and 120 min. Cells were harvested directly into phenol:ethanol and total RNA was purified using Qiagen's RNeasy Mini Kit. Two independent biological experiments were performed for each condition with two technical (dye swap) repeats per biological experiment.
Consent	N/A
Sample/data source location	The University of Sheffield, Sheffield, United Kingdom.

## 1 Direct link to deposited data

The raw data have been deposited in NCBI's Gene Expression Omnibus and are accessible through GEO series accession number GSE55097 (http://www.ncbi.nlm.nih.gov/geo/query/acc.cgi?acc=GSE55097).

## 2 Experimental design, materials and methods

### 2.1 Bacterial culture conditions

Starter colonies of wild-type *E. coli* K-12 MG1655 and *E. coli* K-12 MG1655 *hemA* were grown on nutrient agar and rich broth agar plates [3], respectively, and incubated overnight at 37 °C. For transcriptomic analysis, anaerobic liquid cultures were grown in 250 ml defined medium [3] in mini-fermenter vessels [4] continually sparged with nitrogen, during stirring at 200 rpm. A constant temperature of 37 °C was maintained in the growth vessel using a water jacket from a remote water bath. Cultures were inoculated with 5% v/v of overnight starter cultures grown in rich broth [3] and then harvested and re-suspended in defined medium prior to inoculation. Optical density measurements were made using a Jenway 7315 spectrophotometer.

### 2.2 Preparation of P1 lysates for transduction of the *hemA* mutant allele into *E. coli* strain MG1655

Due to poor growth of the heme-deficient mutant of *E. coli*, the published protocol [5] was adjusted to enable transduction of the *hemA* mutation. Lysates were produced by growing the donor strain (W3110 *hemA*) overnight at 37 °C, during shaking at 200 rpm, in 5 ml rich broth [3] supplemented with 25 μM δ-ALA and 5 mM CaCl_2_. The culture was concentrated to 1 ml in supplemented rich broth and 0.05 ml added to 0.1 ml of the wild-type MG1655 P1 lysate (2 × 10^9^ plaque forming units/ml) diluted as follows: 10^− 5^, 10^− 4^, 10^− 3^ and 10^− 2^. The mix was incubated at 37 °C for 20 min. Pre-warmed terrific broth [TB; tryptone (8 g/l) and NaCl (5 g/l), pH 7] (1 ml; supplemented with 0.5% glucose), 25 μM δ-ALA and 1.5 ml molten terrific broth soft agar (TBSA; TB containing 7 g/l of agar) were added to the bacteria/phage cultures, mixed and poured on top of pre-warmed (37 °C) phage lysate plates (tryptone (8 g/l), yeast extract (5 g/l), NaCl (5 g/l), glucose (2 g/l) and agar (12 g/l), and after autoclaving, 10 ml 0.5 M CaCl_2_, 10 ml 1 M MgCl_2_·6H_2_O and 1 ml 10 mM FeCl_3_ were added) [5]. Once solidified, the plates were incubated at 37 °C in a moist atmosphere until plaques were nearly confluent. The plates were then chilled at 4 °C for 30 min before being overlaid with 5 ml of phage dilution buffer and left overnight at 4 °C. The overlaying liquid was removed and filtered through a sterile 45 μm nitrocellulose filter into a cryovial (Nalgene) and stored at 4 °C under chloroform.

### 2.3 P1 transduction of the recipient strain

An overnight culture of the recipient strain (wild-type *E. coli* MG1655) was grown in 5 ml of TY broth [tryptone (16 g/l), yeast extract (10 g/l) and NaCl (10 g/l)] supplemented with 5 mM CaCl_2_. Aliquots (0.1 ml) of the recipient culture were mixed with 0.1 ml of the W3110 *hemA* lysate at the following dilutions: 10^0^, 10^− 1^ and 10^− 2^. The mix was incubated for 20 min at 37 °C. The entire mixture was plated on rich broth agar [3] supplemented with 0.125 mM Na_4_P_2_O_7_ (an efficient Ca^2 +^ chelator) and spread with 25 μM δ-ALA. After overnight incubation at 37 °C, any putative transductant colonies were restreaked and verified by: 1) streaking a colony onto mutant validation defined medium agar plates containing succinate instead of glucose, with and without δ-ALA, and; 2) cytochrome analysis to confirm cytochrome deficiency.

### 2.4 Sampling and RNA stabilisation

At an OD_600_ of 0.2, a control sample was taken from the untreated cultures of the *E. coli* wild-type and *hemA* mutant strains, immediately followed by the addition of 100 μM CORM-3, or equimolar iCORM-3. Five further samples were taken at 10, 20, 40, 60 and 120 min post-addition of compound. At each time-point, culture samples of 20 ml were removed from minifermenter vessels (anaerobic), added to a chilled mix of 125 μl phenol and 2.38 ml ethanol and vortexed immediately for 5 s, incubated on ice for 5 min followed by centrifugation for 5 min at 5500 rpm, 4 °C.

### 2.5 RNA isolation and determination

RNA was isolated using a Qiagen ‘RNeasy Mini Kit (50)’ according to the manufacturer's instructions. Sample pellets were resuspended in 200 μl of TE buffer containing lysozyme (15 mg/ml) and vortexed for 10 s followed by incubation at room temperature for 5 min. Samples were vortexed every minute during the 5 min incubation. RLT buffer (700 μl) containing β mercaptoethanol (10 μl/ml) was added followed by vigorous vortexing and the addition of 500 μl 96% ethanol. Samples were mixed by gentle swirling and the resultant lysates applied to an RNeasy Mini column, centrifuged for 30 s at 10,000 rpm and the flow-through liquid discarded. Buffer RW1 (350 μl) was added to the column followed by centrifugation for 30 s at 10,000 rpm. After removal of the flow-through liquid, a mixture of 10 μl DNase I with 70 μl buffer RDD was transferred directly onto the RNeasy silicagel membrane and incubated at room temperature for 15 min to allow for DNase digestion. Buffer RW1 (350 μl) was then added to the column followed by a further 5 min incubation at room temperature and centrifugation for 30 s at 10,000 rpm. The column was then washed twice with 500 μl buffer RPE. To elute any remaining ethanol, the column was centrifuged for an additional 30 s at 10,000 rpm. RNase-free water (30 μl) was used for the elution of RNA by centrifugation for 1 min at 10,000 rpm. To increase the yield of RNA, the 30 μl was reapplied to the column and eluted again via centrifugation. The concentration of RNA was determined spectrophotometrically using a Beckman DU 650 UV/Vis spectrophotometer against a DEPC-treated milli-Q H_2_O blank. One A_260_ unit is equal to 40 μg RNA per ml. The quality of the RNA was determined by running the samples on a 0.8% agarose gel in 1 × TBE. Samples producing clear bands corresponding to 16S and 23S species were used for microarray analysis.

### 2.6 cDNA synthesis

RNA (16 μg) for each sample was incubated with 5 μg of random primers (Invitrogen) at 72 °C for 10 min then chilled on ice for 10 min. cDNA synthesis was initiated by the addition of a reaction mix consisting of 6 μl 5 × First Strand (FS) buffer (Invitrogen), 3 μl 0.1 M DTT (Invitrogen), 0.6 μl 50 × dNTP master mix (0.1 mM dATP, dGTP, dTTP and 0.05 mM dCTP) (Roche) and 2.9 μl nuclease-free water (Qiagen). Samples were treated with either 2 μl Cy3 or 2 μl Cy5 (Invitrogen). SuperScript III (1.5 μl 200 U μl^− 1^, Invitrogen) was added to each sample followed by 5 min incubation at 25 °C then an overnight incubation at 50 °C. Samples were hydrolysed by the addition of 15 μl 0.1 M NaOH and incubation at 72 °C for 10 min. To neutralise, 15 μl 0.1 M HCl was added. The samples were cleaned up using a QIAquick PCR purification kit (Qiagen) according to the manufacturer's instructions. Buffer PB was added to the samples at a ratio of 5:1 volumes. The mix was transferred into a spin column and centrifuged for 1 min at 10,000 rpm. The flow-through was discarded and the column was washed twice with 750 μl buffer PE. To ensure removal of all liquids, the column was centrifuged for an additional 1 min at 10,000 rpm. Nuclease-free water (50 μl) was used for the elution of cDNA by centrifugation for 1 min at 10,000 rpm. To measure the concentration of single-stranded cDNA, the labelled cDNA was denatured by heating to 95 °C for 5 min and then quantified using a NanoDrop ND-1000 UV–VIS spectrophotometer version 3.2.1 against a nuclease-free water blank. The following equations were used to calculate the yield of cDNA and its specific activity, respectively.

$$\begin{array}{l} \text{cDNA}\;\left(\mathrm{ng}\right)\;=A260\times 330\;\mathrm{ng}/\mathrm{\mu l}\times 50\;\mathrm{\mu l}\;\text{sample}\;\text{volume}\\ \text{pmol}\;\mathrm{Cy}3\;\text{or}\;\mathrm{Cy}5\;\mathrm{per}\;\mathrm{\mu g}\;\text{cDNA}=\;\left(\left[\mathrm{Cy}3\right]\;\text{or}\;\left[\mathrm{Cy}5\right]\right)/\left(\left[\text{cDNA}\right]\right)\times 1000. \end{array}$$

Samples were suitable for hybridisation if the yield was > 825 ng and the specific activity was > 8 pmol Cy3 or Cy5 per μg DNA.

### 2.7 Hybridisation, washing and scanning procedures

For each reaction, Cy3-labelled cDNA and Cy5-labelled cDNA were diluted in nuclease-free water to give a final concentration of 400 ng in a total volume of 20 μl. The diluted cDNA was boiled at 100 °C for 2 min, chilled on ice for 2 min then incubated at room temperature for a further 2 min. Blocking agent (5 μl of 10 × stock) and 25 μl of 2 × GEx hybridisation buffer HI-RPM were added to each reaction tube and the mix was centrifuged for 1 min at 13,000 rpm. Samples were loaded onto the array slides immediately. The Agilent microarray hybridization assembly consists of an Agilent SureHyb chamber, a gasket slide, an array slide and a clamp. To assemble the chamber, a clean gasket slide was inserted into the Agilent SureHyb chamber base and 40 μl of sample was slowly dispensed onto a gasket well in a ‘drag and dispense’ manner, avoiding contact between the pipette tip or hybridisation solution and the gasket walls. An array slide was placed ‘active side’-down onto the SureHyb gasket slide and the two slides held in place by the SurehHyb chamber cover and the clamp. The assembled chamber was vertically rotated to wet the gasket and the samples were allowed to hybridise for 17 h at 65 °C during gentle rotation.

Following incubation, the microarray wash procedure for Agilent's two-colour platform was carried out according to the manufacturer's instructions. Two gene expression (GE) wash buffers (Agilent), supplemented with 0.005% Triton X-102 (Agilent) prior to first use, were used in this protocol. GE wash buffer #2 was preheated to 37 °C overnight. After hybridisation, the array slides were washed in GE wash buffer #1 at room temperature for 1 min on a stirring platform set to medium speed. The slides were then submerged in GE wash buffer #2 and washed for 1 min then scanned immediately using an Agilent DNA microarray scanner (Agilent Technologies, G2505) controlled by Agilent Scan Control software (v8.5). Output twocolour.tiff image files were produced according to the scanning instructions in the Fairplay III microarray protocol (Agilent Technologies, 252009).

### 2.8 Analysis of microarray data

**Fig. 1:**
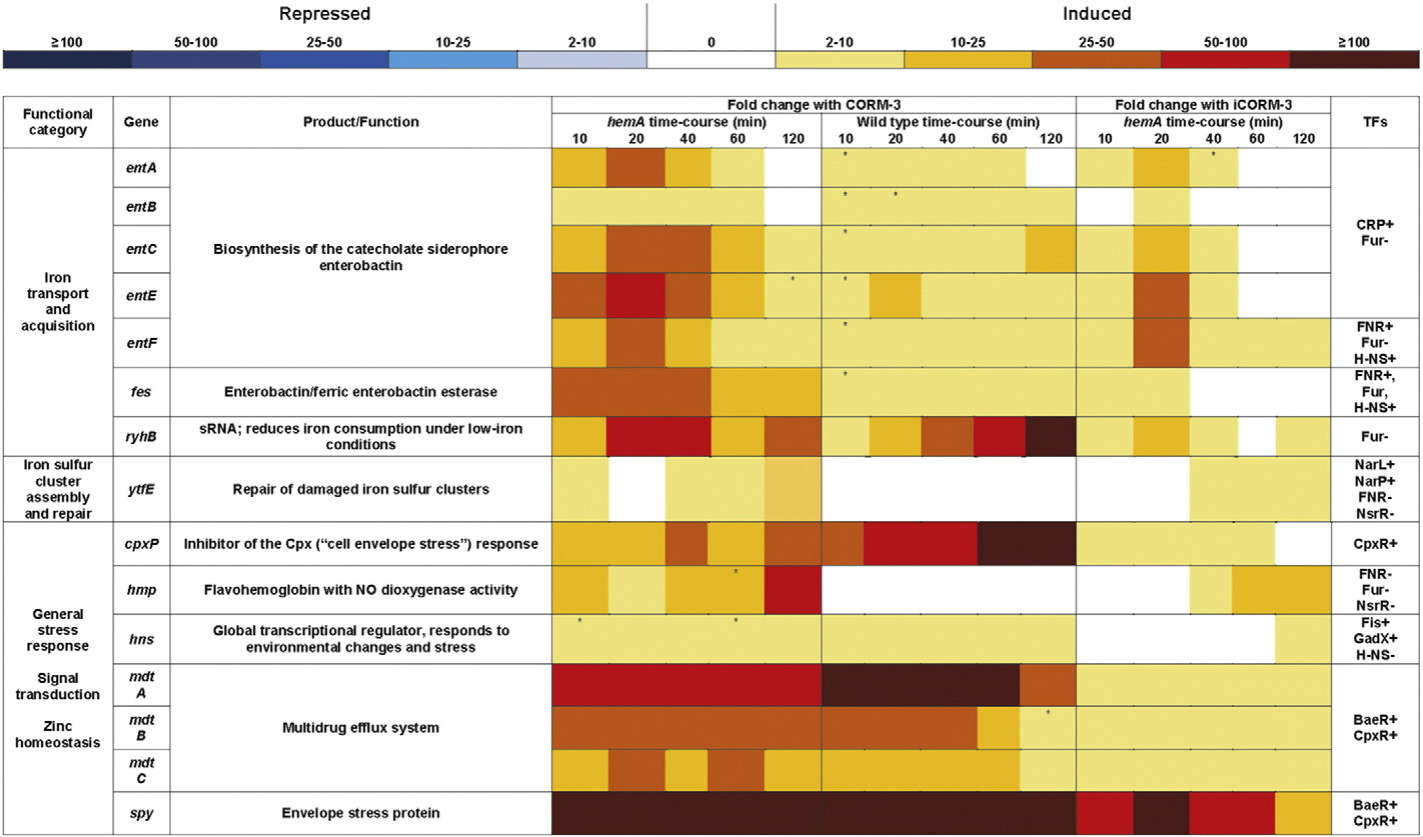
Differential expression of notable genes altered in *hemA* mutant or wild-type bacteria in response to CORM-3 or iCORM-3. The colour-scale bar shows mean fold changes in individual genes of the *hemA* mutant of *E. coli* and the corresponding wild-type grown anaerobically in a defined medium after the addition of 100 μM CORM-3 or, for the mutant only, 100 μM iCORM-3. Unless otherwise stated, *p* values were ≤ 0.05; * indicates a *p* value that exceeds 0.05.

Data acquisition was carried out using Agilent Feature Extraction software (v6.5), which allows measurement of the Cy3 and Cy5 fluorescence of each feature in the scanned microarray image. Data were normalised using GeneSpring GX v7.3 (Agilent Technologies) by dividing the experimental channel by the control channel and applying a global LOWESS normalisation, which removes dye intensity-dependent artefacts caused by non-linearity of Cy5 and Cy3 fluorescence at low levels. Identification of statistically significant gene expression changes was achieved by applying a *t*-test with a 2-fold cut-off and *p* < 0.05. Four replicates were obtained for each condition tested: two biological repeats of CORM-3- or iCORM-3-treated samples hybridised against an untreated control, each with two technical (dye-swap) repeats. Information about gene products and their function was obtained from GeneSpring GX v7.3 (Agilent Technologies). Functional category lists were created using KEGG (Kyoto Encyclopedia of Genes and Genomes) [1], [2]. Relevant regulatory proteins for each gene were identified, where available, using regulonDB and EcoCyc (World Wide Web). The functional categories that contained the most highly altered genes are presented in Fig. 1: differential expression and the function of notable genes within these categories are also shown, along with the transcription factors (TFs) involved in their regulation.

### 2.9 Statistical modelling of transcriptional responses

**Fig. 2:**
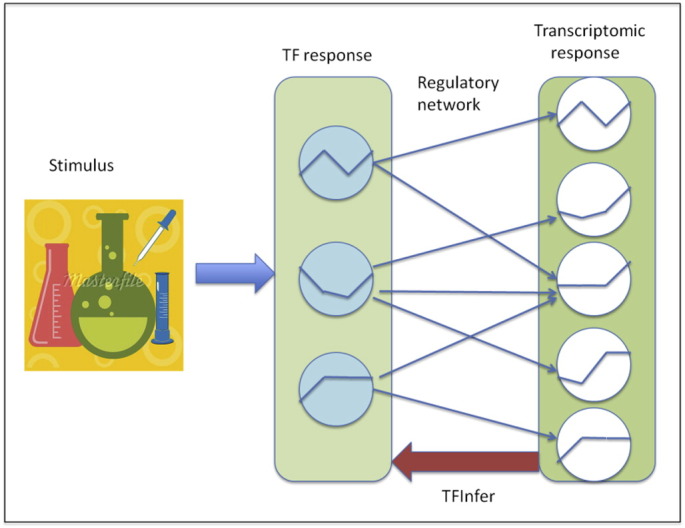
Schematic representation of the TFInfer modelling framework. The conceptual model underpinning TFInfer is that external stimulation elicits transcriptional responses through changes in the activity of transcription factors (TFs). Hence, a stimulus (left-hand side) will determine a change in TF activity (middle layer) which will then result in observable changes in gene expression (right panel). The changes in gene expression depend on the TF activity changes and the wiring diagram of the regulatory network, determining which TF regulates which gene(s). TFInfer adopts a log-linear approximation to model TF-gene interactions in order to solve the inverse problem.

Statistical data modelling was used to infer transcription factor (TF) activities based on the gene expression time-series generated from the microarray analyses. We used a probabilistic model [6], which integrates gene expression data with TF-target information (obtained from data bases such as EcoCyc) to determine the optimal TF activity profiles that can explain the expression data and compatibly with the constraints imposed by the network structure. The model adopts a log-linear approximation, expressing gene expression (log) changes as a weighted linear combination of changes in the activity of the TFs that regulate the genes in the network. A schematic representation of the model is given in Fig. 2; the approach is freely available as open-source software in the TFInfer tool [7]. Although the log-linear approximation is a simplification of the dynamics of transcription, its simplicity permits efficient, large-scale statistical inference, so that one may obtain data-driven estimates of many TF activities simultaneously. The approach has already been extensively adopted for bacterial transcriptomics, leading to numerous novel insights [8].

We then interrogated the results of the TFInfer analysis to deduce differences in TF response between various stimuli. To do so, we computed absolute Pearson correlation coefficients between mean TF profiles from different TFInfer runs, e.g. the *hemA* mutant exposed to CORM-3 versus iCORM-3. For each condition, transcription factor profiles were discarded if a constant time-series could fit within the error bars given by TFInfer, indicating that no significant response could be inferred for that particular TF. Profiles that were discarded from one condition, but not from the other, were given a ‘place-holder’ value of 2. The absolute value of the Pearson correlation coefficient was calculated for the remaining pairs of TF profiles, enabling us to score TFs which behaved consistently/differently in the two conditions. A value between 0 and 1 is given, where a value close to 0 represents a low correlation between transcription factor profiles and a value close to 1 represents transcription factor profiles that are highly correlated. Taking the absolute value means that positive correlations are scored as high as negative correlations. This is done because TFInfer does not know a priori the sign of TF-gene interactions, which means that transcription factor profiles generated from TFInfer could be inverted. Only after the data have been received from the modellers can this be corrected by comparing inferred signs with information from databases on transcription factor activity and flipping a transcription factor profile, as well as the signs of its interactions, where necessary. Flipping a transcription factor profile will not affect the absolute value of the correlation coefficient.

## 3 Conclusions

This work adopted robust and established methods for the preparation and growth of bacterial strains, RNA isolation and microarray analyses, coupled with innovative statistical modelling to identify relevant transcription factors and their roles in bacterial adaptation to CORM-3. Our study showed that CORM-3 is a potent bactericidal molecule, even against bacteria that do not contain heme, evoking general stress responses as well as disrupting the cell membrane, iron acquisition and utilisation mechanisms and zinc management processes.

## Competing interests

The authors declare that there are no competing interests.
